# Supplementary material for: ALK, ROS1, RET and NTRK1–3 Gene Fusions in Colorectal and Non-Colorectal Microsatellite-Unstable Cancers
Source: Int J Mol Sci. 2023 Sep 2;24(17):13610. doi: 10.3390/ijms241713610 (PMC10488195; doi:10.3390/ijms241713610)
Supplement: Supplementary file 1 [file ijms-24-13610-s001.zip › ijms-2583894-supplementary.pdf]

Table S1. MSI occurrence in different cancer types.

Cancer types	Tumors with MSI / Total number of tumors
Colorectal cancers	743/14111 (5.3%)
Gastric cancers	84/1756 (4.8%)
Endometrial cancers	91/506 (18%)
Pancreatic cancers	4/474 (0.8%)
Cervical cancers	7/459 (1.5%)
Ovarian cancers	5/339 (1.5%)
Cholangiocarcinomas	2/64 (3.1%)

Table S2. Evaluation of the fraction of *KRAS/BRAF*-mutated cells in MSI-positive tumors with fusions by droplet digital PCR

ID	Diagnosis	Mutation	Fusion	% drops with mutation in ddPCR	% tumor cells with mutation	% tumor cells by morphologic evaluation
C1274	CRC	*BRAF V600E*	*ETV6::NTRK3(E5;N15)*	17,5%	35%	30%
B4561	CRC	*KRAS A146T*	*ETV6::NTRK2(E5;N15)*	21%	42%	50%
C7527	GC	*KRAS G12C*	*TPM3::NTRK1(T8;N10)*	30,9%	61,8%	30%
C3231	CRC	*BRAF V600E*	*LMNA::NTRK1 (L8;N12)*	5%	10%	30%
D5144	CRC	*KRAS G12D*	*Unbalanced NTRK3*	18%	36%	30%
P29082	CRC	*NRAS Q61K*	*TPM3::NTRK1 (T8;N12)*	22%	44%	No information

Table S3. Clinical characteristics of MSI-positive tumors with gene fusions

		Colorectal cancers		Gastric cancers		Endometrial ceancers	
Total MSI-positive tumors tested for gene fusions		471		69		65	
Gene fusions		positive	negative	positive	negative	positive	negative
Number of samples		58 (12.3%)	413 (87.7%)	4 (5.8%)	65 (94.2%)	3 (4.6%)	62 (95.4%)
Gender	F	33 (56.9%)	223 (54%)	1 (25%)	33(50.8%)		
	M	25 (43.1%)	190 (46%)	3 (75%)	32 (49.2%)		
Age, years	range	22-87	31-86	34-83	41-84	64-71	34-85
	median	62	67	69	66	67	63
	< 40	2 (3.5%)	45 (10.9%)	1 (25%)	0	0	5 (8.1%)
	41 - 50	0	59 (14.3%)	0	9 (13.9%)	0	5 (8.1%)
	51 - 60	11(19%)	79 (19.1%)	0	12 (18.5%)	0	18 (29%)
	61 - 70	27 (45.6%)	144 (34.9%)	1 (25%)	18 (27.7%)	2 (66.7%)	25 (40.3%)
	> 70	18 (31%)	86 (20.8%)	2 (50%)	26 (40%)	1 (33.3%)	9 (14.5%)

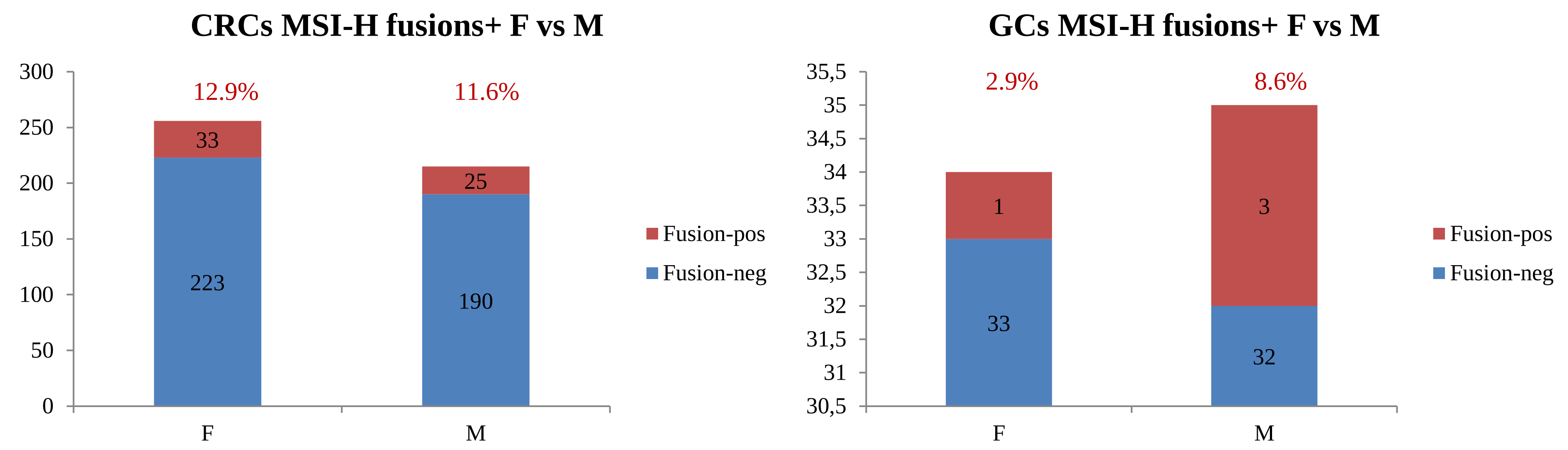

Figure S1. Distribution of fusion-positive tumors according to gender.

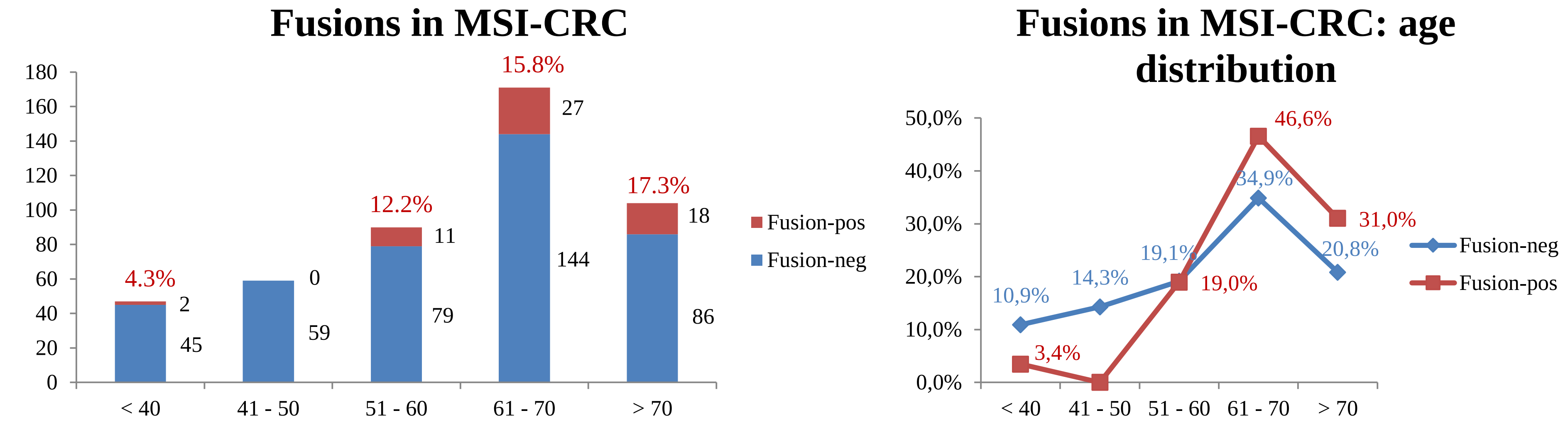

Figure S2. Distribution of MSI-positive CRCs with fusions across various age groups.

Table S4. Frequency of gene rearrangements in MSI-positive CRC patients of different age

	CRC-MSI-H (total)		*CRC-MSI-H* with *KRAS/NRAS/BRAF* mutations		CRC-MSI-H without *KRAS/NRAS/BRAF* mutations	
Age	Cases with fusions / Total number of cases	Statistical significance	Cases with fusions / Total number of cases	Statistical significance	Cases with fusions / Total number of cases	Statistical significance
</=50	2/106 (1.9%)	**p = 0.002**	1/52 (1.9%)	p = 0.976	1/54 (1.9%)	**p = 0.001**
>50	56/365 (15.3%)		4/215 (1.9%)		52/150 (34.7%)	
Total	58/471 (12.3%)		5/267 (1.9%)		53/204 (26%)	

Table S5. PCR primers utilized for MSI detection

Marker	Forward primer sequence	Reverse primer sequence	PCR fragment length, bp
NR21	GAGTCGCTGGCACAGTTCTA	CTGGTCACTCGCGTTTACAA	110
NR24	GCTGAATTTTACCTCCTGAC	ATTGTGCCATTGCATTCCAA	128
NR22	GAGGCTTGTCAAGGACATAA	AATTCTGATGCCATCCAGTT	143
BAT25	TACCAGGTGGCAAAGGGCA	TCTGCATTTTAACTATGGCTC	152
BAT26	CTGCGGTAATCAAGTTTTTAG	AACCATTCAACATTTTTAACCC	182

Table S6. PCR primers and probes used for *ALK/ROS1RET/NTRK1/NTRK2/NTRK3 5'/3'*-end unbalanced expression tests

Multiplex	Target fragment	Oligonucleotide name	Sequence	PCR fragment length, bp
ALK	ALK 5'-fragment (exons 9-10)	ALKex9F	GATGGTGTTGCCTCTCCTC	63
		ALKex10R	CCCCACCATGCGACCATC	
		ALKex9/10P	FAM-ATGTGTCTGACAGGTTCTGGCTG-BHQ1	
	ALK b/p*-fragment (exons 19-20)	ALKex19	TCTCCGGCATCATGATTGTG	73
		ALKex20R	GGGCTCTGCAGCTCCATCT	
		ALKex20P	ROX-ATGGCTTGCAGCTCCTGGTGCT-BHQ2	
	ALK 3'-fragment (exons 22-23)	ALKex22F	GATTTCCTCATGGAAGCCCT	66
		ALKex23R	CCCAATGCAGCGAACAATGT	
		ALKex22/23P	R6G-ATCATCAGCAAATTCAACCACCAG-BHQ2	
ROS1	ROS1 5'-fragment (exons 30-31)	ROS1ex30F	TGGAAGGCTCCATTGAATGTTAA	66
		ROS1ex31R	AGGACCTTGGCTGCATGAA	
		ROS1ex30/31P	FAM-AAACTCATTGTATTTCCACTTCTGTAGCTCAA-BHQ1	
	ROS1 3'-fragment (exons 35-36)	ROS1ex38F	GAGACCTTCTTACTTATTTGCG	71
		ROS1ex39R	GTCAACCAAGGTGAGTAAAGG	
		ROS1ex38/39P	R6G-AGCCCGGATGGCAACGTTTTATG-BHQ2	
RET	RET 5'-fragment (exons 6-7)	RETex6F	AGGAGGGCTCGCCGATTTG	70
		RETex7R	TGATGCCACTGAATGCCTG	
		RETex6/7P	FAM-CAGTTTTCCACACAGACTTTCCC-BHQ1	
	RET 3'-fragment (exons 13-14)	RETex13F	TCATCAAATTGTATGGGGCCT	67
		RETex14R	TATTTGGCGTACTCCACGATG	
		RETex13/14P	R6G-AGCCAGGATGGCCCGCTCCTC-BHQ2	
NTRK1	NTRK1 5'-fragment (exons 3-4)	NTRK1ex3F	GCCTTCCATTTCACTCCTCG	65
		NTRK1ex4R	GAGAGAGACTCCAGAGCGT	
		NTRK1ex3/4P	FAM-TCAGTCGCCTGAATCTCTCCTTCAAC-BHQ1	
		NTRK1ex14F	GCTCATGGTCTTTGAGTATATGCG	75
		NTRK1ex15R	AGCTTGGCATCAGGTCCATG	

	NTRK1 b/p*-fragment (exons 14-15)	NTRK1ex14/15P	JOE-ATCGGAGGAAGCGGTTGAGGTC-BHQ1	
	NTRK1 3'-fragment (exons 16-17)	NTRK1ex16F	ACCTACGGCAAGCAGC	80
		NTRK1ex17R	CGCTCCAACTCACGTCC	
		NTRK1ex16/17P	ROX-AACACGGAGGCAATCGACTGCAT-BHQ2	
NTRK2	NTRK2 5'-fragment (exons 11-12)	NTRK2ex11F	AATGAAATCCCTTCCACAGACG	88
		NTRK2ex12R	CCACCACAGACGCAATCAC	
		NTRK2ex11/12P	FAM-AACATCTCTCGGTCTATGCTGTGGT-BHQ1	
	NTRK2 b/p*-fragment (exons 15-16)	NTRK2ex15F	GAATGCTATAACCTCTGTCCTGAG	97
		NTRK2ex16R	CACGGTGGAAGTCCTTGC	
		NTRK2ex15/16P	ROX-TTCAGGGTCTTCACTGCCACCAAGAT-BHQ2	
	NTRK2 3'-fragment (exons 17-18)	NTRK2ex17F	ACGTGTACAGCACTGACTACTAC	104
		NTRK2ex18R	GCTTTCCGTCGTGAATTTCCT	
		NTRK2ex17/18P	JOE-ACACAATGCTGCCCATTCGCTGGAT-BHQ1	
NTRK3	NTRK3 5'-fragment (exons 13-14)	NTRK3ex13F	GGTTCTCTTCGTCATGATCAAC	86
		NTRK3ex14R	CCTCCTCACCACTGATGAC	
		NTRK3ex13/14P	FAM-TTTGGAATGAAGGGTCCCGTGGCT-BHQ1	
	NTRK3 b/p*-fragment (exons 14-15)	NTRK3ex14F	CCAGTACTTCCGTCAGGGAC	93
		NTRK3x15R	GTCTCTCCTCTTAATGTGCTGC	
		NTRK3ex14/15P	ROX-AACTGCCACAAGCCGGACACGTATG-BHQ2	
	NTRK3 3'-fragment (exons 16-17)	NTRK3ex16F	AATACATGAAGCATGGAGACC	68
		NTRK3ex17R	CACAAGGATCATTGCATCTGG	
		NTRK3ex16/17P	JOE-CATGGGCCCTGAGGAACTTATT-BHQ1	

*b/p - breakpoint

Table S7. PCR primers and probes for the detection of common *ALK* translocations (multiplex reaction)

Fusion	Breakpoint	Oligonucleotide name	Oligos for 5'-companions	Oligonucleotide name	Oligos for 3'-companion	PCR fragment length, bp
EML4::ALK	*E13;A20*	EML4ex13F	TGGAGCAAAACTACTGTAGAG	ALKex20r	GGGCTCTGCAGCTCCATCT	101
	E20;A20	EML4ex20F	CTAACTCGGGAGACTATGAAAT	ALKex20P	FAM-ATGGCTTGCAGCTCCTGGTGCT-BHQ1	85
	E6;A20	EML4ex6F	CATAAAGATGTCATCATCAACCA			80
	E18;A20	EML4ex18F	ACACAGACGGGAATGAACAG			100

Table S8. PCR primers and probes for the detection of common *ROS1* translocations (multiplex reaction)

Fusion	Breakpoint	Oligonucleotide name	Oligos for 5'-companions	Oligonucleotide name	Oligos for 3'-companion	PCR fragment length, bp
CD74::ROS1	*C6;R34*	CD74ex6F	CCACTGACGCTCCACCGAA	ROS1ex32R	TACTCCCTTCTAGTAATTTGG	65
SDC4::ROS1	*S2;R32*	SDC4ex2F	ACCAGACGATGAGGATGTAG	ROS1ex32P	R6G-ATGCCTGGTTTATTTGGGACTC-BHQ2	99
	S4;R32	SDC4ex4F	CCGTTGAAGAGAGTGAGGAT			141
SLC34A2::ROS1	*SLC4;R32/R34*	SLC34A2ex4F	GGATTGGGAGATTGATTTTACT	ROS1ex34R	ACAACCAGAALNAATATTCCAACTA	138/154
	SLC13;R32/34	SLC34A2ex13F	CATTAGCAGAGAGGCTCAG	ROS1ex34P	FAM-CTTGTTTCTGGTATCCAAAATCA-BHQ1	68/84
EZR::ROS1	*E10;R34*	EZRex10F	AGACAAAGAAGGCAGAGAGA			85
FIG::ROS1	*F7;R35*	FIGex7F	GTTTGTACCTTGATGAGTTAGA	ROS1ex35R	TGTCACCCCTTCCTTGGCA	134
TPM3::ROS1	*T8;R35*	TPM3ex8F	GAAAAGACAATTGATGACCTG	ROS1ex35P	FAM-TCTGGCATAGAAGATTAAGAATC-BHQ1	75
LRIG3::ROS1	*L16;R35*	LRIG3ex16F	GGATGGGTACGTGTCTTCAG			142

Table S9. PCR primers and probes for the detection of common *RET* translocations (multiplex reaction)

Fusion	Breakpoint	Oligonucleotide name	Oligos for 5'-companions	Oligonucleotide name	Oligos for 3'-companion	PCR fragment length, bp
CCDC6::RET	*C1;RE12*	CCDC6ex1F	GCAAAGCCAGCGTGACCAT	RETex12R	TAGAGTTTTTCCAAGAACCAAG	74
KIF5B::RET	*K15;RE12*	KIF5bex15F	GCTGTGGGAAATAATGATGTAA	RETex12P	FAM-CTTCCGAGGGAATTCCCACTTTG-BHQ1	78
	K16;RE12	KIF5Bex16F	AGGAGTTAGCAGCATGTCAG			89
	K18;RE12	KIF13Aex18F	TGCAAACCTCAGTGCCAA			75
	K22;RE12	KIF5Bex22F	CTTTGTTCAGGACCTGGCTA			88
	K23;RE12	KIF5Bex23F	ATAATCTTGAACAGCTCACTAAA			89
	K24;RE12	KIF5bex24F	GTCAAAGAATATGGCCAGAAG			95
NCOA4::RET	*N7;RE12*	NCOA4ex7F	AGGCTGTATCTCCATGCCA			79
	N8;RE12	NCOA4ex8F	AGCAGACCTTGGAGAACAGT			77
RELCH::RET	*R10;RE12*	RELCHex10F	CATTGTTCCCAATGTGCTATT			88
KIAA1217::RET	*KIAA11;RE12*	KIAA1217ex11F	CTTTCCTCCTGCGTCAAG			99

Table S10. PCR primers and probes for the detection of *BCR::PKHD1* and *CLIP1::LTK* translocations

BCR ::PKHD1 and *CLIP1::LTK* multiplex						
Fusion	Breakpoint	Oligonucleotide name	Oligos for 5'-companions	Oligonucleotide name	Oligos for 3'-companion	PCR fragment length, bp
BCR ::PKHD1	*B8;P36*	BCRex8F	GAGGAGATCACACCCCGAC	PKHD1ex36R<br>PKHD1ex36P	AGCTGTGAGTCCTGGACCAT<br>FAM-TAAAGAAAAGTTGCCCTCTCCCTTC-BHQ1	97
CLIP1::LTK	*C17;L11*	CLIP1ex17F	GAGGAGCTGAGAAAGCAAG	LTKex11R<br>LTKEX11P	GCTCTGAGCAGAGTAACATTG<br>HEX-TTGGCCCGGCCCAGTCCT-BHQ2	118

Table S11. PCR primers and probes for the detection of rare *ALK* translocations

Fusion	Breakpoint	Oligonucleotide name	Oligos for 5'-companions	Oligonucleotide name	Oligos for 3'-companion	PCR fragment length, bp
EML4::ALK	*E2;A20*	EML4ex2F	CTGAAGATCATGTGGCCTCA	ALKex20R	GGGCTCTGCAGCTCCATCT	108
KIF5B::ALK	*K17;A20*	KIF5Bex17F	CGATGCCCTCAGTGAAGAAC	ALKex20P	FAM-ATGGCTTGCAGCTCCTGGTGCT-BHQ1	96
KLC1::ALK	*KCL9;A20*	KLC1ex9F	CTGAAGATCATGTGGCCTCA			96
DCTN1::ALK	*D26;A20*	DCTN1ex26F	CTGGTCTCTGGCATTGCTG			77
SQSTM1::ALK	*SQ5;A20*	SQSTM1ex5F	TGAAGAACGTTGGGGAGAGT			100

Table S12. PCR primers and probes for the detection of rare *RET* translocations

Fusion	Breakpoint	Oligonucleotide name	Oligos for 5'-companions	Oligonucleotide name	Oligos for 3'-companion	PCR fragment length, bp
Rare *RET* translocations multiplex 1						
CUX1::RET	*C10;R12*	CUX1ex10F	AGATCCAGAAGGCACCA	RETex12R	TAGAGTTTTTCCAAGAACCAAG	77
FYCO1::RET	*F8;R12*	FYCO1ex8F	GCTGAAATCATGGACTACCA	RETex12P	FAM-CTTCCGAGGGAATTCCCACTTTG-BHQ1	87
ITGA8::RET	*I30;R12*	ITGA8ex30F	ACCGACAGGGAACAGC			108
KIF5B::RET	*K19;R12*	KIF5Bex19F	GTAGAAGCAAAAGCAAAACTTAT			92
MPRIP::RET	*M19;R12*	MPRIPex19F	CACAGGGCAAGGATGC			83
RELCH::RET	*R10;R12*	RELCHex10F	CATTGTTCCCAATGTGCTATT			88
SLC39A8::RET	*S6;R12*	SLC39A8ex6F	TCCATTTTGATAATGTCAGTGT			89
TRIM33::RET	*T14;R12*	TRIM33ex14F	CAGGAGGAGTGCTTGCA			73
ZBTB41::RET	*Z9;R12*	ZBTB41ex9F	AGGCAAATCAAGTCTGGAA			95
Rare *RET* translocations multiplex 2						
ADD3::RET	*A1;R12*	ADD3ex1F	AGGGAGGGGAAACACAA	RETex12R	TAGAGTTTTTCCAAGAACCAAG	91
ANKS1B::RET	*A1;R12*	ANKS1Bex1F	AGCCCTCGCGGATC	RETex12P	FAM-CTTCCGAGGGAATTCCCACTTTG-BHQ1	97
CCDC186::RET	*C10;R12*	CCDC186ex10F	TACAGGAGCAGCTTCAAA			73
FRMD4A::RET	*F12;R12*	FRMD4Aex12F	GTATGACTACCATGATAAAGTGAA			88
KIAA1217::RET	*K11;R12*	KIAA1217ex11F	CTTTCCTCCTGCGTCAAG			99
KIF13A::RET	*K18;R12*	KIF13Aex18F	AACCTCAGTGCCAATAGGAA			75
MYO5C::RET	*M25;R12*	MYO5Cex25F	TCACTTCTGATGGCTTGAA			107
RASSF4::RET	*R3;R12*	RASSF4ex3F	AGGGCAAGAGCTTCCA			86
TBC1D32::RET	*T9;R12*	TBC1D32ex9F	GTTGATACCAAGGCTGTGT			90
WAC::RET	*W3;R12*	WACex3F	ACAGAGTTAGAGAGAGGGATGGT			78

Rare *RET* translocations multiplex 3						
CCNYL2::RET	*C6;R12*	CCNYL2ex6F	CATATTTGATGAACAGTTACACC	RETex12R	TAGAGTTTTTCCAAGAACCAAG	85
LSM14A::RET	*L9;R12*	LSM14Aex9F	GAGTTTGCGGATTTTGAATATAG	RETex12P	FAM-CTTCCGAGGGAATTCCCACTTTG-BHQ1	78
PCM1::RET	*P29;R12*	PCM1ex29F	GCAATTATGAAAGAAGTCATTCC			87
PRKG1::RET	*P7;R12*	PRKG1ex7F	GCAATTATGAAAGAAGTCATTCC			93
PTPRK::RET	*P3;R12*	PTPRKex3F	GCAGTGAGCACCTTTTG			87
RUFY2::RET	*R9;R12*	RUFY2ex9F	TTAATGAAAACACAGCAGCA			81
SIRT1::RET	*S8;R12*	SIRT1ex8F	GGCAAAGGAGCAGATTAGTA			86
SLC25A36::RET	*S1;R12*	SLC25A36ex1F	CGCTGGTGCATCTGTT			79
SORBS1::RET	*S8;R12*	SORBS1ex8F	ATCCCAACCTTCCTTCTGA			83
TSSK4::RET	*T1;R12*	TSSK4ex1F	TTCCTGCCCCGTGAA			75

Table S13. PCR primers and probes for the detection of common *NTRK1* translocations

Fusion	Breakpoint	Oligonucleotide name	Oligos for 5’-companions	Oligonucleotide name	Oligos for 3’-companion	PCR fragment length, bp
NTRK1 translocations multiplex 1						
LMNA::NTRK1	*L2;N11/12*	LMNAex2F	CTCAGTGAGAAGCGCACG	NTRK1ex10R	GGTGTTTCGTCCTTCTTCTCC	103/112
	L3;N11	LMNAex3F	ACTTCCAGAAGAACATCTACAGTG	NTRK1ex10P	FAM-ACACTAACAGCACATCTGGAGACCCG-BHQ1	69
	L4;N10/12	LMNAex4F	GCTGGAGAAGACTTATTCTGCC	NTRK1ex11R	ATGAAATGCAGGGACATGGC	74/77
	L8;N12	LMNAex8F	CCACCAAAGTTCACCCTGAAG	NTRK1 ex11P	FAM-TCTCGGTGGCTGTGGGCCTG-BHQ1	91
	L10;N11	LMNAex10F	TGAGGATGGAGATGACCTGC	NTRK1ex12R	GGAAGAGGCAGGCAAAGAC	77
	L11;N12	LMNAex11F	TACCTCCTGGGCAACTCC	NTRK1ex12P	FAM-CTGTGCTGGCTCCAGAGGATGGG-BHQ1	85
NTRK1 translocations multiplex 2						
BCAN::NTRK1	*B12;N10*	BCANex12-F	CAACTACCACCTGTCCTACAC	NTRK1ex10R	GTGTTTCGTCCTTCTTCTCCA	86
				NTRK1 ex10P	FAM-CACTAACAGCACATCTGGAGACC-BHQ1	
NTRK1 translocations multiplex 3						
IRF2::NTRK1	*I1;N8/10*	IRF2ex1F	AGGCAGGTTGTTGGGTTTC	NTRK1ex8R	ATCCACAGAGAAGGGGATGC	118
		IRF2ex1P	FAM-ACACTAACAGCACATCTGGAGACCCG-BHQ1	NTRK1ex10R	GGTCTCCAGATGTGCTGTTAG	74
NTRK1 translocations multiplex 4						
TPM3::NTRK1	*T8;N10/12*	TPM3ex8F	GAGATCGGTAGCCAAGCTG	NTRK1ex9R	CACCGGCGAGAAGGAGA	76
	T7;N10	TPM3ex7F	CAAGATTCTTACTGATAAACTCAAGGAG			92/96
		TPM3ex7P	JOE-CTTCCAGGTCATCAATTGTCTTTTCCAGC-BHQ1	NTRK1ex10R	GTGTTTCGTCCTTCTTCTCCA	
	T9;N10	TPM3ex9F	CTGGACCACGCCCTCAATG	NTRK1ex10P	FAM-CACTAACAGCACATCTGGAGACC-BHQ1	80
	T10;N9	TPM3ex10F	ATGGATTTAGTACAGGTTACTCAGG	NTRK1ex12R	ATGAAATGCAGGGACATGGC	
		TPM3ex10P	FAM-AAGGAATTTAATCTTGTTCAGCTTGAGGAG-BHQ1	NTRK1ex12P	FAM-CTGTGCTGGCTCCAGAGGATGGG-BHQ1	110

Table S14. PCR primers and probes for the detection of common *NTRK2* translocations

NTRK2 translocations multiplex 1						
Fusion	Breakpoint	Oligonucleotide name	Oligos for 5'-companions	Oligonucleotide name	Oligos for 3'-companion	PCR fragment length, bp
VCL::NTRK2	*V16;N12*	VCLex16F	TGGAAACATTTCCGACCCTG	NTRK2ex12R	GGCAAAATCCCACCACAGA	63
AFAP1::NTRK2	*A13;N12*	AFAP1ex15F	TCGGGCTCAACTCGCA	NTRK2ex12P	FAM-GTCTATGCTGTGGTGGTGATTGCGTCT-BHQ1	61
VCAN::NTRK2	*VN6;N12*	VCANex6F	GCAGATTTGATGCCTACTGC	NTRK2ex14R	GGGCTGGCAGAGTCATC	70
NACC2::NTRK2	*NC5;N13*	NACC2ex5F	GGTCCTGAACGCTGTGAAA	NTRK2ex14P	FAM-TCATCATCATTGCTGATAACGGAGGCT-BHQ1	111
NOS1AP::NTRK2	*NO9;N13*	NOS1APex9F	GCTGTCAGGACAGAACGC	NTRK2ex15R	GGCTCCTTCGCCTAGCT	111
TBC1D2::NTRK2	*TB6;N14*	TBC1D2ex6F	GGGAAGATAGAGCACCTGAAG	NTRK2ex15P	FAM-ATGTTATGTCGCTTGATGTGCTGAACAA-BHQ1	64
TRIM24::NTRK2	*TR12;N15/16*	TRIM24ex12F	CAGTCACCAAATTCATCAGTGC	NTRK2ex16R	GAGGTTGGTCAGGAGCTCG	99/86
TRAF2::NTRK2	*TF9;N15*	TRAF2ex9F	CGCATACCCGCCATCTTC	NTRK2ex16P	FAM-TGAAGGATGCCAGTGACAATGCA-BHQ1	84
SQSTM1::NTRK2	*SQ4;N15*	SQSTMex4F	CCCCACGGCAGAATCAG	NTRK2ex17R	GGTTGCCCTCAGCCATC	76
	SQ5;N15/N17	SQSTMex5F	TCCGAGTGTGAATTTCCTGAAG	NTRK2ex17P	FAM-ACACGGCCCTGATGCCGT-BHQ1	121/103
NTRK2 translocations multiplex 2						
Fusion	Breakpoint	Oligonucleotide name	Oligos for 5'-companions	Oligonucleotide name	Oligos for 3'-companion	PCR fragment length, bp
AGBL4::NTRK2	AG6;N16	AGBL4ex6F	CTGACGATAACCAGCCCTG	NTRK2ex12R	GGCAAAATCCCACCACAGA	65
STRN3::NTRK2	ST7;N16	STRN3ex7F	GAGAAGCACGGAGTTCGG	NTRK2ex12P	FAM-GTCTATGCTGTGGTGGTGATTGCGTCT-BHQ1	82
WNK2::NTRK2	W24;N16	WNK2ex24F	GCGGCAGCTCAAGGTC	NTRK2ex14R	GGGCTGGCAGAGTCATC	78
GKAP1::NTRK2	G9;N16	GKAP1ex9F	GATGCTGAAATCCAGAAGCTG	NTRK2ex14P	FAM-TCATCATCATTGCTGATAACGGAGGCT-BHQ1	91
KCTD8::NTRK2	K1;N16	KCTD8ex1F	AGCAGCTACACCGAGTACA	NTRK2ex15R	GGCTCCTTCGCCTAGCT	74
PRKAR2A::NTRK2	PR2;N16	PRKAR2Aex2F	CAGTTCCTAGCAGATTTAATAGACG	NTRK2ex15P	FAM-ATGTTATGTCGCTTGATGTGCTGAACAA-BHQ1	79
PAN3::NTRK2	P1;N17	PAN3ex1F	ACTCGATGGACCGCGG	NTRK2ex16R	GAGGTTGGTCAGGAGCTCG	64
STRN::NTRK2	S3;N16	STRNex3F	TGAAGCCTCCAAGCTATGATTC	NTRK2ex16P	FAM-TGAAGGATGCCAGTGACAATGCA-BHQ1	70
				NTRK2ex17R	GGTTGCCCTCAGCCATC	
				NTRK2ex17P	FAM-ACACGGCCCTGATGCCGT-BHQ1	

Table S15. PCR primers and probes for the detection of common *NTRK3* translocations

Fusion	Breakpoint	Oligonucleotide name	Oligos for 5'-companions	Oligonucleotide name	Oligos for 3'-companion	PCR fragment length, bp
NTRK3 translocations multiplex 1						
ETV6::NTRK3	*E4;N14*	ETV6ex4F	CATTCTTCCACCCTGGAAACTC	NTRK3ex13R	GCAAGTCCAACTGCTATGGATAC	90
		ETV6ex4P	FAM-TTCTGATGCAGTATGACCTCCGGCTGT-BHQ1	NTRK3ex14R	CACTGATGACAGCCACGG	
	E5;N13/N15	ETV6ex5F	CTGGCTTACATGAACCACATCA	NTRK3ex15R	CCTCACCCAGTTCTCGCTT	99/130
		ETV6ex5P	FAM-TGCTATTCTCCCAATGGGCATGGCGT-BHQ1			
	E6;N14/N15	ETV6ex6F	CCGGATAGTGGATCCCAAC			70/103
		ETV6ex6P	FAM-TTATGGTTTCCCCACAGTCGAGCCAGT-BHQ1			
NTRK3 translocations multiplex 2						
EML4::NTRK3	*E2;N13/N14*	EML4ex2F	CTTTGGCTGATGTTTTGAGGC	NTRK3ex13R	GCAAGTCCAACTGCTATGGATAC	101
		EML4ex2P	FAMTGAGACTGATTTTTTCACTGAGGCCACBHQ1	NTRK3ex14R	CACTGATGACAGCCACGG	99
